# Antibody–Drug Conjugates for Multiple Myeloma: Just the Beginning, or the Beginning of the End?

**DOI:** 10.3390/ph16040590

**Published:** 2023-04-14

**Authors:** Upasana Ray, Robert Z. Orlowski

**Affiliations:** 1 Department of Lymphoma/Myeloma, The University of Texas MD Anderson Cancer Center, 1515 Holcombe Blvd., Houston, TX 77030-4009, USA; 2 Department of Experimental Therapeutics, The University of Texas MD Anderson Cancer Center, 1515 Holcombe Blvd., Unit 429, Houston, TX 77030-4009, USA

**Keywords:** multiple myeloma, antibody–drug conjugate, relapsed and refractory disease

## Abstract

Multiple myeloma is a malignancy of immunoglobulin-secreting plasma cells that is now often treated in the newly diagnosed and relapsed and/or refractory settings with monoclonal antibodies targeting lineage-specific markers used either alone or in rationally designed combination regimens. Among these are the anti-CD38 antibodies daratumumab and isatuximab, and the anti-Signaling lymphocytic activation molecule family member 7 antibody elotuzumab, all of which are used in their unconjugated formats. Single-chain variable fragments from antibodies also form a key element of the chimeric antigen receptors (CARs) in the B-cell maturation antigen (BCMA)-targeted CAR T-cell products idecabtagene vicleucel and ciltacabtagene autoleucel, which are approved in the advanced setting. Most recently, the bispecific anti-BCMA and T-cell-engaging antibody teclistamab has become available, again for patients with relapsed/refractory disease. Another format into which antibodies can be converted to exert anti-tumor efficacy is as antibody–drug conjugates (ADCs), and belantamab mafodotin, which also targets BCMA, represented the first such agent that gained a foothold in myeloma. Negative results from a recent Phase III study have prompted the initiation of a process for withdrawal of its marketing authorization. However, belantamab remains a drug with some promise, and many other ADCs targeting either BCMA or other plasma cell surface markers are in development and showing potential. This contribution will provide an overview of some of the current data supporting the possibility that ADCs will remain a part of our chemotherapeutic armamentarium against myeloma moving forward, and also highlight areas for future development.

## 1. Introduction

**Figure 1:**
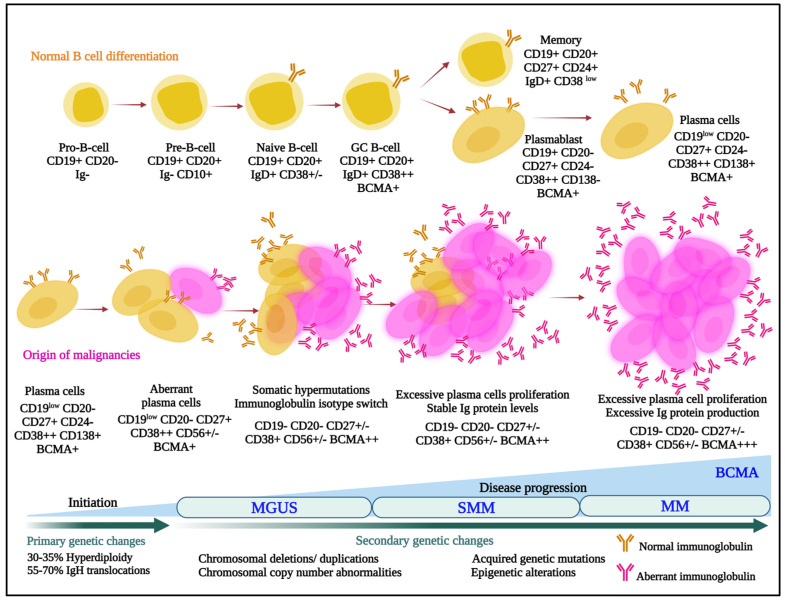
Normal (upper panel) and aberrant (lower panel) B-cell differentiation processes are outlined, and cells are annotated with some of the lineage-specific surface markers that may be targets for antibody–drug conjugates. MGUS, monoclonal gammopathy of undetermined significance; SMM, smoldering multiple myeloma; MM, multiple myeloma; BCMA, B-cell maturation antigen; IgH, immunoglobulin heavy locus.

Multiple myeloma is the second most commonly diagnosed hematologic malignancy in most developed countries. It is characterized by infiltration and poorly controlled proliferation of malignant plasma cells in the bone marrow, and sometimes in extramedullary sites as well. These cells represent the terminally differentiated versions of pro-B-cells that originate in the bone marrow and develop to transitional cells. Next, they traffic to lymph nodes, where they are exposed to antigens and become plasmablasts, and, finally, return to the marrow as plasma cells (Figure 1) [1].

In that myeloma cells retain many characteristics of plasma cells, they typically continue to secrete intact immunoglobulins (Igs) such as IgG or IgA, and rarely IgD or IgE, or immunoglobulin fragments, including either free light or heavy chains. Notably, this characteristic was leveraged by Georges Köhler and César Milstein when they fused murine myeloma cells with mouse spleen cells from an immunized donor to form hybridomas [2], which allowed mass production of monoclonal antibodies. This earned them the 1984 Nobel Prize in Physiology or Medicine, and ushered in a new era, especially starting in 1997, when the anti-CD20 monoclonal rituximab became the first approved antibody for cancer therapy. Uncontrolled secretion of monoclonal Igs in myeloma also serves as a quantifiable biomarker for disease burden. Unfortunately, these Igs can also be pathogenic and contribute to organ dysfunction, such as of the heart and kidneys, especially in the setting of light chain (AL) amyloidosis [3]. Notably, outcomes for patients with myeloma have been improving dramatically [4,5,6,7,8] with the advent of novel therapies [9], and especially of immunomodulatory agents (IMiDs), proteasome inhibitors (PIs), anti-CD38 antibodies, and B-cell maturation antigen (BCMA)-targeted drugs. However, even patients with newly diagnosed myeloma do not uniformly enter complete remissions. Also, those that do still develop relapsed and relapsed/refractory disease that is characterized by shorter remission durations with each subsequent therapy, and myeloma remains incurable in the vast majority [10,11,12]. This supports the presence of a persistent unmet medical need in several areas spanning the spectrum of myeloma, and the possibility that new drug categories with new mechanisms of action could still play a significant role in the treatment of patients. Moreover, the stepwise process through which plasma cells develop leads to enhanced expression and exposure of lineage-specific cell surface proteins that can serve as targets for antibody-based therapeutics (Figure 1). This fact, along with the flexibility of the antibody–drug conjugate (ADC) platform, suggests that ADCs could play a substantial role in myeloma therapy. Our task will be to provide an overview of what has been accomplished to date, and highlight possible future promising directions, with a focus on those ADCs that either have been or are in clinical trials or are about to enter the clinic.

## 2. Rationale for ADCs in Myeloma

### 2.1. Mechanisms of Action of ADCs against Cancer

**Figure 2:**
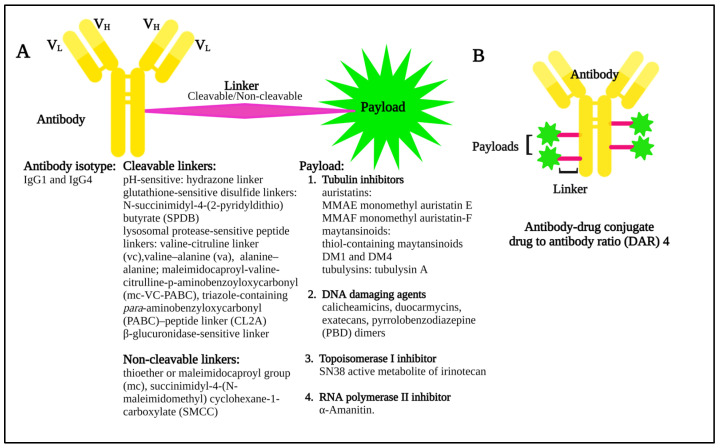
(**A**) Diagram showing the basic components of an antibody–drug conjugate, including some of the possible linker and payload combinations. (**B**) Model structure of an antibody–drug conjugate with a drug to antibody ratio (DAR) 4.

Many cell surface molecules are subject to internalization through phagocytosis, pinocytosis, or receptor-mediated endocytosis, the last of which can be mediated by mechanisms dependent on clathrin or caveolae, or that are independent of both [13]. These pathways provide portals of entry into the cell interior that can be leveraged by ADCs, which are broadly structured to include an antibody to target the cell of interest, a linker, and a payload, or warhead of interest (Figure 2) that induces cytotoxicity. 

**Figure 3:**
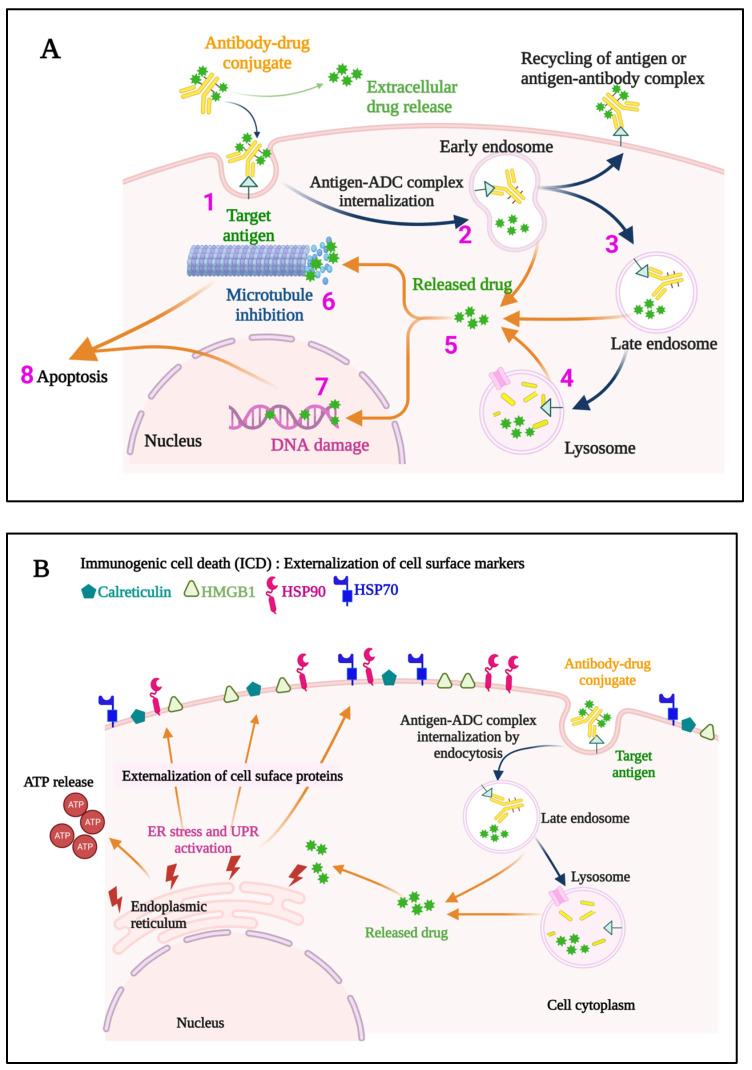
A representation of the events occurring both at the cell membrane and intracellularly that contribute to the direct cytotoxic mechanisms of action of an ADC (**A**) is provided. The ADC binds to the specific target antigen on the cell surface (1), the ADC-antigen complex is internalized (2), and endosome/lysosome mediated processing (3,4) results in the release of the cytotoxic payload into the cytosol (5). If an anti-microtubule agent is the payload, aberrant spindle formation may occur causing cell cycle arrest and apoptosis (6), while DNA intercalators cause DNA damage (7), ultimately leading to apoptosis of the cell (8). Some ADCs also induce immunogenic cell death (ICD) (**B**), which elicits a host immune anti-tumor response. Hallmarks of ICD include externalization of Calreticulin, and Heat shock protein 90 (HSP90) and HSP70, externalization and later release of High mobility group box 1 (HMGB1), and release of ATP. ER, endoplasmic reticulum; UPR, unfolded protein response; ADC, antibody–drug conjugate.

Once internalized, ADCs release their payloads and, in the case of anti-microtubule agents, for example, these can induce cell cycle arrest, resulting ultimately in the triggering of apoptosis (Figure 3A). Indeed, the story of the Greek Trojan Horse is often used as an analogy to describe the means by which ADCs enter cells and exert this portion of their mechanism of action. Additional anti-tumor mechanisms leveraged by ADCs include induction of immunogenic cell death (ICD) (Figure 3B), and Fc-mediated effector functions, including antibody-dependent cellular cytotoxicity (ADCC), phagocytosis (ADCP), and complement-dependent cytotoxicity (CDC). The modular design of ADCs (Figure 2) and the multiple options available for each component confer a high level of flexibility to this class of drugs. A full discussion of these options is beyond the scope of our work, and the interested reader is thus referred to several recent excellent reviews in these areas [14,15]. Briefly, however, these include using antibody isotypes or modifications to enhance or decrease/delete Fc-mediated effector functions, linkers that are cleavable or non-cleavable, and even acid-labile for release into the tumor microenvironment (TME), and a variety of drug classes targeting different mechanisms [14,15].

### 2.2. Why ADCs Should Be Good Therapeutics against Myeloma

Other than the strong case for ADCs as anti-cancer therapies in general, the rationale in myeloma is based in part on the availability of lineage-specific markers that can be used to identify plasma cells (Figure 1). Techniques have been established and validated that allow for identification of neoplastic plasma cells, such as the so-called EuroFlow next generation flow cytometry [16]. When this is used for minimal residual disease testing, it involves multi-parameter flow to detect CD19, CD27, CD38, CD45, CD56, CD81, CD117, CD138, and cytoplasmic Igλ and Igκ. Notably, the ability to differentiate between normal and neoplastic plasma cells could theoretically enhance the therapeutic index of an ADC-based approach. In addition, the Fc gamma receptor 2B (FcɣRIIB) is expressed on plasma cells, and may provide another portal of ADC entry into myeloma cells [17], thereby potentially enhancing cytotoxicity and highlighting why ADCs could be especially effective against myeloma. However, internalization through FcɣRs has been described as a mechanism for off-target toxicity of ADCs, especially when these are aggregated, in cells that express these receptors [18]. Moreover, soluble FcɣRs may be increased in myeloma with the stage of the disease [19] and could theoretically protect myeloma cells by binding to ADCs and thwarting this route for internalization. Finally, disease progression in myeloma is often associated with clonal evolution and the development of genetically complex and heterogeneous subclones that follow different pathways through time and space [20,21,22,23,24], but these often retain stable surface proteomes [25] that can be targeted multiple times. The latter is in part borne out by the remarkably high and deep response rates achieved by BCMA-directed CAR T-cell therapies, providing a roadmap for the optimization of other cell surface-targeted therapies such as ADCs.

## 3. Clinical Progress in Leveraging ADCs against Myeloma

The first clinical trial of an ADC against myeloma, which is now of more historical interest, utilized the CD19-targeted anti-B4 antibody linked to blocked ricin and was based on the rationale that CD19 may be present on myeloma clonogenic cells [26,27]. Out of five patients treated, no responses were seen, and toxicities included transaminase elevations, myalgias, thrombocytopenia, nausea, vomiting, neurologic toxicity, decreased performance status, and capillary leak syndrome. Modern studies have targeted some of the now more widely accepted myeloma cell surface markers (Figure 1), and the relevant data are reviewed below for these antigens and the various agents either previously or currently in development.

### 3.1. Targeting BCMA

BCMA, also known as CD269 or Tumor necrosis factor receptor superfamily member 17, has been one of the most popular targets for ADCs, and for CAR T-cells and bispecific T-cell engagers. This is in part due to its high expression on neoplastic plasma cells and late mature B-cells [28], and the prominent role of B-cell activating factor and A proliferation-inducing ligand-mediated activation of BCMA in promoting myeloma cell survival and therefore BCMA-dependence in the TME [29].

#### 3.1.1. AMG 224

This Amgen-developed ADC is an IgG1 anti-human BCMA ADC conjugated with mertansine (DM1), an anti-tubulin maytansinoid through a non-cleavable linker (Table 1), which therefore relies on lysosomal degradation of the antibody for drug release. A completed Phase I study targeted relapsed/refractory myeloma patients who had received a median of 7 prior lines of therapy (LOT). AMG 224 was administered every three weeks intravenously at doses ranging from 30 to 300 mg [30]. Common Grade 3 or higher adverse events (AEs) included thrombocytopenia and anemia, and ocular treatment-emergent adverse events (TEAEs) were seen. These included dry eyes (in 7%), and increased lacrimation, conjunctival hemorrhage, diplopia, eye irritation or pruritus, blurred vision, reduced visual acuity, visual impairment, and vitreous hemorrhage in 3% each. Other AEs of note included infusion-related reaction, fatigue, transaminitis, musculoskeletal pain, and myalgias. Among 40 patients who received at least one drug dose, the objective response rate was 23%, and while the median duration of response (DOR) in the dose escalation was 14.7 months, these data led Amgen to discontinue development of AMG 224. Notably, of patients with available serum samples for soluble BCMA (sBCMA) testing, responders tended to have lower sBCMA levels. 

#### 3.1.2. Belantamab Mafodotin (Previously GSK2857916)

Belantamab mafodotin (BelaMaf), a humanized IgG1 anti-BCMA monoclonal antibody conjugated to the microtubule inhibitory drug monomethyl auristatin F (MMAF) (Table 1), was the first and, so far, only ADC to receive regulatory approval for myeloma from the Food and Drug Administration. In addition to inducing direct cytotoxic cell death after the release of MMAF, which inhibits microtubule polymerization and induces G2/M arrest and apoptosis, BelaMaf activated ADCC and ADCP [25,31], as well as ICD [32]. A Phase I trial targeted patients whose myeloma had progressed after prior stem cell transplantation, alkylators, PIs, and IMiDs, and administered this ADC as an intravenous infusion once every three weeks. This identified a dose of 3.40 mg/kg as the recommended Phase II dose (RP2D) based on safety, with Grade 3 and 4 toxicities including anemia and thrombocytopenia, and corneal toxicity being the other main adverse event [33,34], which was predominantly of Grade 1 or 2 severity. Preferred AE terms for the corneal toxicities seen in more than one patient included blurred vision, dry eyes, photophobia, and visual impairment. Detailed ophthalmologic examination described that these were associated with corneal microcyst-like epithelial changes following BelaMaf internalization leading to epithelial cell apoptosis [35]. These changes were reversible once the apoptotic cells were replaced with new corneal epithelial cells. In a second part of Phase I that included patients treated at the RP2D, 21/35 (60%) had a response, defined as an at least 50% reduction in biochemical parameters of disease burden, which translated into a median progression-free survival (PFS) of 12 months and a median DOR of 14.3 months. Baseline sBCMA levels were also compared with myeloma responses as measured by reduction in serum and/or urine monoclonal proteins and/or serum free light chain concentrations, but no clear relationship was reported. 

These encouraging data prompted a Phase II, registration-enabling trial known as DREAMM-2, which enrolled almost 200 patients who were randomized to a lower dose of 2.5 mg/kg and the previously identified 3.4 mg/kg [36]. Notably, a more advanced patient population was enrolled in this trial since patients were required to have suffered disease progression after three or more LOT, and their myeloma had to be refractory to both IMiDs and PIs, and either refractory or intolerant (or both) to an anti-CD38 antibody. Indeed, the median number of prior LOT for the 2.5 mg/kg cohort was 7, and this was 6 for the 3.4 mg/kg cohort. Perhaps not unexpectedly, therefore, a lower ORR was seen, which was 31% and 34% in the two cohorts, while the median PFS was 2.9 and 4.9 months, respectively. The most common Grade 3–4 AEs were keratopathy in 21% and 27% of patients at the lower and higher doses, respectively, and anemia and thrombocytopenia were also described. Thrombocytopenia occurred at a median time to onset of 22 days in 35% of patients who received the 2.5 mg/kg dose, and in 58% of patients at the 3.4 mg/kg dose level. Anemia was also commonly observed and seen in 24% and 37% of patients at the 2.5 mg/kg and 3.4 mg/kg doses, respectively. Finally, Grade 1–2 infusion-related reactions were observed with the first infusion in 18% (2.5 mg/kg dosing) and 15% (3.4 mg/kg dosing) of patients, respectively. Due in part to the paucity of other available agents for so-called triple-class refractory (TCR) disease, defined as having progressed through prior PIs, IMiDs, and anti-CD38 monoclonals, BelaMaf was approved on 5 August 2020 [37]. 

Subsequent reports of so-called real-world experiences with BelaMaf have suggested similar to perhaps improved patient outcomes compared to DREAMM-2. For example, Vaxman et al. noted a response rate of 33% but a median PFS and overall survival (OS) of only 2 and 6.5 months, respectively, even though some patients received BelaMaf-based combinations [38]. However, this cohort was generally more heavily pre-treated, with a median of 8 prior LOT, and included patients who had received CAR T-cell-based treatment. In contrast, Atieh et al. [39] noted an ORR of 43% and a median PFS and OS of 4.9 and 10.7 months, respectively, in 35 TCR patients with a median of 5 prior LOT. Similarly, Shragai et al. [40] reported an ORR of 45.5% and a median PFS and OS of 4.7 and 14.5 months, respectively, in 106 patients with a median of 6 prior LOT. 

Current research into BelaMaf has focused on two central aspects, the first being to reduce the incidence of ocular toxicities, which is an AE that is seen with other ADCs as well [41]. Consensus guidelines recommend eye examinations before and during each cycle, and also upon any worsening of symptoms, close collaboration between hematologist/oncologists and eye care professionals to grade toxicities, liberal use of preservative-free eye drops, and dose and/or schedule modifications [42]. Equally of note, a subgroup analysis of DREAMM-2 [43] suggested that 88% of patients (14/16) who had a clinical response and a prolonged treatment delay (>63 day) nonetheless maintained or even deepened their response. This suggested that lower and/or less frequent BelaMaf dosing may maintain clinical efficacy, and it has prompted studies of such approaches. However, a smaller, observational study suggested that BelaMaf dose reduction or discontinuation was associated with progression within 3 months [44], which may undermine this hypothesis. 

A second area of focus for ongoing studies (Table 2) has been on combining BelaMaf with other anti-myeloma agents to enhance its efficacy and, potentially, to allow less frequent dosing and mitigate toxicities. For example, one single institution experience of BelaMaf with dexamethasone in 35 TCR patients [39] reported an ORR of 43%, and a median PFS and OS of 4.9 and 10.7 months, respectively. Formal clinical trials are underway with many partner agents, such as in the DREAMM-3 study [45], which included a cohort with the gamma-secretase inhibitor nirogacestat, which should enhance cell surface BCMA expression [46] and could act synergistically with BelaMaf. While final results have not appeared in the peer-reviewed literature, a preliminary report suggested the possibility of enhanced efficacy based on an increase in the number of patients with a stringent complete response (sCR) or CR with nirogacestat and BelaMaf [47]. Another interesting combination from DREAMM-3 could be BelaMaf with bortezomib and dexamethasone, which showed a 78% ORR in relapsed and/or refractory myeloma patients with a median of 3 prior LOT [48]. In addition, BelaMaf on four different doses and schedules is being combined with lenalidomide and dexamethasone as part of the DREAMM-6 trial, an interim report of which focused on 45 patients with a median of 3 prior LOT and the majority (58%) having had prior lenalidomide [49]. The ORR ranged from 42% when BelaMaf was given at 1.9 mg/kg every 8 weeks to 75% when this same dose was given on an every four-week schedule, though a robustly powered and randomized study would be needed to confirm the possibility that the latter was superior. BelaMaf’s role in activating ICD informed a study combining it with the checkpoint inhibitor pembrolizumab, and a recent report of the primary analysis focused on 34 patients with a median of 5 prior LOT [50]. This regimen demonstrated an ORR of 47%, with most (10/16) responders having an at least VGPR, while the DOR was 8.0 months and the median PFS was 3.4 months. Finally, BelaMaf with pomalidomide and dexamethasone (Pd) could be especially attractive as well, with an ORR of 86.0% in 50 response-evaluable triple-class exposed patients with a median of 3 prior LOT, of whom 72% were TCR, while the median PFS was 15.6 months [51]. 

Beyond the relapsed and/or refractory setting, BelaMaf is also being evaluated as part of induction therapy for newly diagnosed symptomatic myeloma. For example, one Phase I/II study is examining transplant-ineligible patients receiving BelaMaf at 1.4 to 2.5 mg/kg given every 8 weeks with lenalidomide and dexamethasone [52]. Among 36 patients in the analysis, a preliminary ORR of 97.2% is being reported, including 52.8% of patients achieving a very good partial remission (VGPR), defined as an at least 90% disease burden reduction, and no episodes of progressive disease were noted. A second study is evaluating transplant-eligible patients receiving BelaMaf at 2.5 mg/kg given every 8 weeks with bortezomib, lenalidomide and dexamethasone (VRd) using an every four-week VRd schedule [53]. Among 40 patients with a median follow-up of 6 months, the PFS has been 89.3% and the ORR was 82.1% after four cycles, with VGPR or better seen in 69.2% and all CR patients had achieved minimal residual disease (MRD) negativity. BelaMaf is also being studied in multiple other myeloma settings, including as a maintenance therapy and in smoldering disease (Table 2).

Finally, BelaMaf may be promising in patients with relapsed/refractory AL amyloidosis, a plasma cell dyscrasia that can be detected in the absence or presence of myeloma, based on two early reports. Zhang et al. reported on six patients with a median of 6 prior LOT who received standard of care BelaMaf [54]. Five (83%) achieved a hematologic response, including three with a CR, and cardiac responses were seen in all but one patient (80%) with baseline cardiac involvement. Encouraging also was a report from Khwaja et al. detailing outcomes in eleven AL patients with a median of 3 prior LOT and a median of two organs involved [55]. An ORR of 64% was noted, and 55% of patients experienced a VGPR or CR, while the PFS at a median follow-up of 7.1 months was 83%. These findings prompted an ongoing Phase II study of BelaMaf within the European Myeloma Network for this population, and a preliminary report is indicating a best hematologic response rate of 52.9% among seventeen patients with a median of 3 prior LOT [56]. Organ responses are being seen as well, with 29.4% of patients seeing any organ response at 3 months, and the median follow-up of 8.4 months suggests that this figure could improve further. One other study of BelaMaf in AL amyloidosis is currently planned, as is a study for plasmablastic lymphoma, which also is often BCMA-positive.

#### 3.1.3. HDP-101

Amanitin is an agent that is being investigated as a warhead for ADCs by Heidelberg Pharma, and functions through the potent and specific inhibition of RNA polymerase II [57] (Table 1). In pre-clinical models, HDP-101 showed efficacy against both proliferating and resting myeloma cells [58], potentially providing a mechanism to attack quiescent myeloma stem-like or myeloma-initiating cells. Intriguingly, in vivo experiments documented cures in some animals after just a single dose of drug, reflecting potentially the strong potency of the amanitin-based warhead. Moreover, other studies suggested that it may show greater potency than BelaMaf [59], and preferential activity against high-risk deletion 17p models, in part due to co-deletion producing haploinsufficiency of RNA polymerase IIA [60]. Clinically, this agent is in a first-in-human (FIH) trial (Table 2) using an adaptive Bayesian logistic regression model with overdose control principle to guide dose escalation. A preliminary report on the first four patients treated with a median of 11 prior LOT indicated that the initial two cohorts were well tolerated without DLTs, and specifically with no keratopathy [61].

#### 3.1.4. MEDI2228

MEDI2228 is a BCMA-targeted ADC that leverages pyrrolobenzodiazepine dimers in its warhead (Table 1), which are released after proteolytic linker cleavage in lysosomes and produce highly cytotoxic DNA crosslinks [62]. Consistent with this mechanism of action, pre-clinical studies of MEDI2228 have shown potent activation of DNA damage responses (DDR) in myeloma models, and synergy with DDR inhibitors targeting ATM/ATR/WEE1 checkpoint kinases [63] as well as bortezomib. Interestingly, MEDI2228 also has shown synergy with daratumumab through interferon-driven immune responses and enhanced CD38 expression [64]. In a Phase I dose-escalation study, MEDI2228 was administered intravenously every 3 weeks to 82 relapsed/refractory myeloma patients who had received 2–11 prior LOT, and 0.14 mg/kg was identified as the maximum tolerated dose (MTD) [65]. Notable TEAEs at this level included photophobia (53.7%), thrombocytopenia (31.7%), rash (29.3%), increased gamma-glutamyltransferase (24.4%), dry eyes (19.5%), and pleural effusion (19.5%). The ORR in this cohort was 61.0% and included 10 patients (24.4%) who achieved a VGPR, and a median DOR was not reached. Interestingly, BCMA expression was examined by immunohistochemistry, and there was no difference in the bone marrows of responders compared with non-responders. Despite these interesting findings, a press release indicated that AstraZeneca decided to halt further development of this ADC.

### 3.2. Targeting CD38

CD38, or Cluster of differentiation 38, is a type II membrane glycoprotein highly expressed on plasma cells that encodes a bifunctional enzyme having both ADP-ribosyl cyclase and cyclic ADP-ribose hydrolase activities [66]. As CD38 is not expressed on pluripotent hematopoietic progenitor cells and normal resting lymphocytes, this therefore provides potential for a therapeutic index. 

#### 3.2.1. STI-6129

STI-6129, also known as LNDS1001, is a fully human anti-CD38 antibody conjugated to Duostatin 5.2, an MMAF-derived microtubule inhibitor [67] (Table 1). Pre-clinical studies showed that it induced CD38-dependent cytotoxic activity in vitro without homotypic aggregation of tumor cells, as is seen with daratumumab, and broad efficacy in multiple xenograft models. Several clinical trials of STI-6129 are recruiting patients, including one for relapsed/refractory myeloma and another for AL amyloidosis (Table 2).

#### 3.2.2. MT-0169

MT-0169, previously known as TAK-169, is a second generation engineered toxin body composed of a single-chain variable fragment with an affinity for CD38 that is fused to the enzymatically active de-immunized Shiga-like toxin-A subunit (Table 1). In pre-clinical studies, this agent induced irreversible ribosome inactivation and potent cytotoxicity against CD38-positive human myeloma models [68]. Among tested primary samples, TAK-169 was equally effective against plasma cells from patients with newly diagnosed and relapsed/refractory myeloma, though a fraction of cells from daratumumab-refractory patients were less sensitive [69]. A very preliminary report of the Phase I study in four patients with relapsed/refractory myeloma and at least 5 prior LOT noted one TEAE of asymptomatic Grade 2 reversible myocarditis and Grade 3 troponin elevation [70], raising concern about its further development.

#### 3.2.3. TAK-573

While not a classical ADC in that it does not have a directly cytotoxic warhead, TAK-573, also known as Modakafusp alfa, is a first-in-class, humanized, anti-CD38, IgG4 antibody genetically fused to two attenuated interferon alpha-2b molecules. This agent has shown direct anti-proliferative activity against myeloma cell lines pre-clinically, and also combined well with standard of care agents in vivo, potentially in part through modulation of immune responses [71]. In an ongoing Phase I/II study, TAK-573 is being administered as a 1–4-h intravenous infusion at 10 different dose levels and on 4 different schedules, and a dose expansion was opened at 1.5 mg/kg every 4 weeks when anti-myeloma activity was seen [72]. Among 30 patients on this dose and schedule, frequent Grade 3–4 TEAEs included neutropenia (63%), thrombocytopenia (43%), leukopenia (40%), anemia (30%), and lymphopenia (30%), while other notable Grade 3/4 events included 1 bleeding event and 3 infections. An ORR of 40% has been reported despite the fact that this cohort had a median of 7 prior LOT, and 37% of patients who were previously anti-CD38 refractory responded, while the median PFS was 6 months, and the median DOR has not been reached. This prompted initiation of a broader development effort in myeloma, with several studies either planned or underway.

### 3.3. Targeting CD46

CD46, a type I membrane protein that is a negative regulator of the complement system, was felt to be an especially attractive target for 1q amplified patients since this gene is located at cytogenetic band 1q32.2 [73], whose amplification is associated with an adverse prognosis [74]. Notably, CD46-targeted ADCs did indeed show anti-proliferative activity pre-clinically against myeloma cell lines and primary samples, and in an orthometastatic xenograft model [73]. In part with this rationale, Fortis Therapeutics advanced FOR46, which was an MMAF-based ADC, into the clinic for relapsed or refractory myeloma. Unfortunately, while 31 participants were enrolled into the Phase I study, no safety or efficacy data have been reported, and the study is now listed as having been terminated on clinicaltrials.gov.

### 3.4. Targeting CD48

CD48, or Signaling lymphocyte activation molecule family member 2 (SLAMF2), is a glycosylphosphatidylinositol-anchored membrane protein expressed on myeloma cells that participates in activation and differentiation pathways. SGN-CD48A, an ADC linked to MMAE, was examined in fourteen myeloma patients in a Phase I trial, but no safety or efficacy data have been reported, and the study has been terminated according to clinicaltrials.gov.

### 3.5. Targeting CD54

CD54, also known as Intracellular adhesion molecule 1 (ICAM1), or BB2, is a transmembrane glycoprotein of the immunoglobulin superfamily that is aberrantly overexpressed in several cancers, including myeloma [75]. A CD54 ADC has been developed by Sherbenou et al., who conjugated the anti-ICAM1 M10A12 IgG1 to MMAF and showed it was effective in vitro and in vivo [76]. However, clinical translation of CD54-targeted ADCs may be challenging considering the prior experience with unconjugated CD54 antibodies such as BI-505. This agent initially showed good safety in relapsed/refractory myeloma patients from a Phase I study that did not identify an MTD [77], but a second study in smoldering myeloma showed no activity [78], and its development was later stopped due to an adverse cardiovascular event.

### 3.6. Targeting CD56

CD56, also known as Neural cell adhesion molecule 1, is aberrantly expressed on transformed plasma cells in up to 80% or more of patients and used in the assessment of MRD negativity by multi-parameter flow [79], suggesting it is a valid therapeutic target. Lorvotuzumab mertansine (IMGN901) is an ADC based on the anti-CD56 antibody N901 to which the maytansinoid DM1 was added with a disulfide linker (Table 1). Pre-clinical studies demonstrated that IMGN901-depleted CD56-positive myeloma cells from mixed cultures reduced serum paraprotein secretion, inhibited tumor growth, and increased survival in a murine model [80]. While a number of single-agent and combination clinical trials were completed, final data have been published only from the Phase I single-agent study, which enrolled 37 relapsed patients into the dose-escalation portion [81]. The toxicity profile was felt to be favorable, with the only Grade 3 or 4 AE reported in more than one patient being fatigue. Unfortunately, among the 35 response-evaluable patients, a PR was noted in two (5.7%), while an additional four (11.4%) had minor responses, defined as between a 25% and 50% reduction in disease burden, and further development has therefore halted. 

### 3.7. Targeting CD74

CD74 is a non-polymorphic type II transmembrane glycoprotein that functions as a major histocompatibility complex (MHC) class II chaperone, and participates in other non-MHC protein trafficking, such as of angiotensin II type I receptor [82]. Moreover, it serves as the high-affinity receptor for macrophage migration inhibitory factor, which may play a role in myeloma pathobiology [83]. Since it is highly expressed on B-cells and plasma cells, CD74 has been seen as a valid myeloma target [84].

#### 3.7.1. Milatuzumab Doxorubicin

Milatuzumab doxorubicin, also known as hLL1-DOX and IMMU-110, was developed as an ADC for myeloma in part because it showed efficacy in pre-clinical models [85], and since a prior Phase I trial of unconjugated milatuzumab did not show efficacy [86]. A Phase I study of the ADC was performed, and 17 patients were enrolled, but neither safety nor efficacy results have been reported, and its clinicaltrials.gov record indicates that this trial was terminated. 

#### 3.7.2. STRO-001

STRO-001 from Sutro Biopharma is a fully human aglycosylated anti-CD74 antibody with a site-specific non-cleavable linker-maytansinoid warhead generated through a novel cell-free protein synthesis technology (Table 1). Pre-clinical studies confirmed its potent efficacy against myeloma cell lines and primary cells, and in in vitro and in vivo models [87]. A Phase I study targeting patients with non-Hodgkin lymphoma or myeloma is underway, and preliminary results of the lymphoma cohort have been presented [88]. Treatment emergent adverse events (TEAEs) occurring in ≥20% of patients included chills, fatigue, nausea, anemia, headache, pyrexia, infusion reaction, decreased appetite, and abdominal pain. One dose limiting toxicity (DLT) included a Grade 3 thromboembolic event, while no ocular toxicities were noted, and the clinical benefit/disease control rate was 25% (4/16). Notably, Sutro Biopharma is also producing CC-99712, a BCMA-targeted ADC conjugated through a non-cleavable linker to a maytansinoid with a drug:antibody ratio (DAR) of 4, which is in Phase I testing through Celgene and Bristol-Myers Squibb.

### 3.8. Targeting CD319

SLAMF7, also known as CD319 and previously as CD2 subset 1, is a validated myeloma target bound by elotuzumab. This agent is approved in combination with both lenalidomide and dexamethasone [89], as well as Pd [90], in the relapsed and/or refractory setting, providing a rationale for ADCs to target CD319. ABBV-838 has just such an ADC, made from a humanized recombinant IgG1κ antibody to SLAMF7 attached through a valine-citrulline linker to monomethyl auristatin E (MMAE, Table 1). The FIH Phase I trial included 75 patients who received at least one dose, with the most common TEAEs being neutropenia and anemia, fatigue, and nausea, while 16% of patients developed corneal deposits [91]. An ORR of 10.7% was reported, including VGPR in 2 (2.7%) and PR in 6 (8.0%) of patients, which did not support its further development.

### 3.9. Targeting CD138

CD138, also known as Syndecan-1, is a type I transmembrane proteoglycan cell adhesion molecule overexpressed on transformed plasma cells that promotes angiogenesis, tumor growth and metastases, and adhesion to the TME [92,93], making it a viable target. 

#### Indatuximab Ravtansine

nBT06, a murine/human chimeric CD138-specific antibody conjugated with highly cytotoxic maytansinoid (DM4) derivatives (Table 1), initially showed anti-myeloma efficacy in pre-clinical models [94]. Prominent features of its mechanism of action included induction of G2/M cell cycle arrest, followed by apoptosis with cleavage of caspases, and blockade of adhesion to bone marrow stromal cells. On translation to the clinic for relapsed and/or refractory myeloma patients previously treated with an IMiD and a PI, BT062 was administered on two different schedules that showed MTDs ranging from 140 to 160 mg/m^2^ [95]. Most AEs were Grade 1 or 2, with the most common being diarrhea and fatigue, and over 75% of pretreated patients achieved stable disease or better on a once every 3-week schedule, while minor and partial responses occurred in 14.7% with a median PFS of 3 months and OS of 26.7 months on the more intensive, weekly schedule. More recently, combination study results were reported in which indatuximab ravtansine was given with lenalidomide/dexamethasone (IrRd) or pomalidomide/dexamethasone (IrPd) [96]. Common Grade 3–4 AEs for both were neutropenia, anemia, and thrombocytopenia, while TEAEs leading to discontinuation occurred in 35/64 (55%), including five patients (8%) with a fatal TEAE, though these were not attributed to the drug. Objective responses for IrRd were noted in 33/46 patients (71.7%), and in 12/17 (70.6%) who received IrPd. 

### 3.10. Targeting CD307

Fc-receptor-like 5 (FCRL5), also known as FCRH5 and CD307, is a member of the immunoglobulin receptor superfamily and the Fc-receptor-like family that may be involved in B-cell development and differentiation and is expressed on malignant plasma cells (Table 1). DFRF4539A, an anti-FcRH5 ADC linked to MMAE, was evaluated in a Phase I study in relapsed/refractory myeloma, and while Grade 3 anemia was the most common TEAE, there were two episodes of Grade 3 renal failure [97]. In part as a result of these, the MTD was not reached, and DFRF4539A demonstrated limited activity with only 2 PRs (5%) as the best responses. 

## 4. Future Directions for ADCs in Myeloma

Despite an abundance of targets available on the myeloma cell surface, the history of ADC-based drug development in multiple myeloma has not been encouraging so far, with many failures and, indeed, only one drug, BelaMaf, reaching a regulatory approval. Of further concern is the competition provided by two commercially available CAR T-cells targeting BCMA, including idecabtagene vicleucel [98] and ciltacabtagene autoleucel [99], and the BCMA bispecific teclistamab [100]. All three of these have substantially higher overall response rates and response durations, and while randomized trials are formally needed to make such comparisons, the results of such efforts would not seem to be in doubt. This could be further borne out by a comparison of cevostamab, a CD305-targeted bispecific which is showing robust efficacy and durability, to the history of the CD305-targeted ADC, which showed marginal efficacy. Finally, even BelaMaf is now in question since GlaxoSmithKline initiated the process for withdrawal of its US marketing approval based upon the outcome of the DREAMM-3 Phase III trial. In this open-label randomized head-to-head superiority study, 325 patients were randomized in a 2:1 ratio to receive BelaMaf at 2.5 mg/kg every 3 weeks or Pd. The observed median PFS was longer for BelaMaf compared to Pd at 11.2 versus 7 months, respectively, and the VGPR or better rate was better with BelaMaf than Pd, at 25% versus 8%, respectively. However, this PFS represented a hazard ratio of only 1.03 (95% confidence interval of 0.72–1.47), indicating a negative trial. Do these and other arguments indicate that this is the beginning of the end for the utility of ADCs in myeloma?

Fortunately, all is not lost for ADCs for myeloma in general, and this is true for BelaMaf as well. With regard to the latter, considering the excellent ORR of 86.0% with BelaMaf in combination with Pd and the median PFS of 15.6 months noted earlier [51], it seems likely that a Phase III trial comparing Pd to BelaMaf with Pd would be positive. In addition, the ongoing DREAMM-8 Phase III study comparing BelaMaf/Pd to bortezomib/Pd has an estimated completion date of 31 March 2023, and since the latter regimen previously conferred a PFS of 11.2 months [101], it is possible that positive data from this trial could lead to full approval of BelaMaf.

Moreover, looking beyond lineage-specific targets expressed on plasma cells of all myeloma patients to those that may be expressed in molecularly defined subsets could be a productive line of research. For example, Fibroblast growth factor receptor 3 (FGRF3) is frequently overexpressed and sometimes activated in patients with the t(4;14) translocation [102]. However, the Phase I study of the FGFR3-targeted ADC LY3076226, which showed an acceptable safety profile, did not enroll any myeloma patients [103]. Thus, it could be of interest to revisit this agent which, if active, would be an especially exciting development considering that t(4;14) myeloma is considered a high-risk subtype with an inferior prognosis [104].

Beyond better clinical trial design, reducing the toxicities of ADCs could allow patients to receive more consistent therapy with a better quality of life and potentially improved long-term outcomes. Ocular toxicities are clearly a challenge for several ADCs and, interestingly, pre-clinical studies have suggested that the mechanism responsible may be their uptake into corneal epithelial cells through macropinocytosis, resulting in inhibition of cellular proliferation [105]. Importantly, since uptake was facilitated by positive charges or hydrophobic residues on the surface of macromolecules, the authors found that decreasing ADC hydrophobicity by attaching polyethylene glycol moieties reduced cytotoxicity, suggesting possible future approaches. Hematologic toxicities in general, and thrombocytopenia in particular, can similarly be a challenge in some patients. In this regard, some studies have described a role for FcɣR-mediated uptake of microtubule-targeted ADCs into megakaryocytes, producing a disrupted cytoskeletal structure [106] and inhibition of megakaryocyte maturation [107] as possible contributors. Thus, ADC re-engineering to avoid this impact could be of benefit in some cases, such as by using IgG4 isotypes, or IgG1 isotypes with decreased/deleted Fc-mediated effector functions as backbones that would not engage FcɣRs. However, this risks a loss of ADCC, ADCP, and CDC mechanisms that IgG1 antibodies can engage, and should be considered only with caution if these mechanisms substantially add to the anti-tumor activity of a specific ADC.

Improved antibody design will likely also play a critical role in improving the standing of ADCs in myeloma moving forward. For example, optimizing the binding affinity of an antibody to a myeloma cell surface target may enhance anti-tumor efficacy [108]. Moreover, optimization of the drug conjugate for the biology of myeloma may be needed as many ADCs have so far employed anti-microtubule agents, and yet myeloma is not classically considered to be a highly proliferative malignancy [109,110]. Thus, agents that do not rely on brisk cell cycle progression, such as amanitins, or that are more potent, such as pyrrolobenzodiazepine dimers, may prove to be superior. Finally, studies aimed at obtaining a better understanding of the determinants of ADC internalization into myeloma cells specifically, as well as factors that determine ADC sensitivity and resistance [111], will be critical. Some patients have tolerated ADCs well and derived substantial long-term benefits, and our ability to ideally identify patients who are most appropriate for CAR T-cell-based therapies is only beginning to emerge [112]. Moreover, initial data in the post-BelaMaf space suggest that patients previously treated with this ADC who then progress suffer from neither a significant loss of BCMA expression nor from significant immune impairment [113], while BCMA loss has been described in at least some patients who have received CAR T-cell-based therapies [114,115]. This suggests the possibility that BelaMaf or other such ADCs could be rationally used earlier in the disease course without compromising the later use of bispecifics or CAR T-cells. Ultimately, if we can identify those patients who are less likely to benefit from CAR T-cells and bispecifics and are likely to show sensitivity with good tolerability to optimized ADCs, then this class of drugs will truly make a significant contribution to our therapeutic armamentarium against myeloma. Therefore, we could indeed be just at the beginning of understanding how best to leverage the exciting power of ADCs against myeloma.

## Figures and Tables

**Table 1:** Antibody–Drug Conjugates in Clinical Trials in Multiple Myeloma.

SL No.	ADCs	IgG Subtype	Payload	Linker	Manufacturer
1	AMG 224 (anti-BCMA–MCC–DM1)	IgG1	DM1 is a semi-synthetic derivative of the ansamycin antibiotic maytansine	MCC: noncleavable linker 4-(N-maleimidomethyl) cyclohexane-1-carboxylate conjugated to lysines in the antibody	Amgen Inc. Thousand Oaks, CA, U.S.
2	Belantamab mafodotin (anti-BCMA-mcMMAF)	IgG1κ	MMAF is the microtubule inhibitor monomethyl auristatin F	mc: non-cleavable, protease-resistant maleimidocaproyl linker	GlaxoSmithKline Brentford, United Kingdom
3	HDP-101 (anti-BCMA-amanitin conjugate)	IgG1	Amanitin is a bicyclic peptide from the death cap mushroom and a potent and specific inhibitor of RNA polymerase II	Cleavable linker/site-specific	Heidelberg Pharma AG Munich, Germany
4	MEDI2228 (anti-BCMA-pyrrolobenzodiazepine (PBD) conjugate)	IgG1	PBD dimer is a DNA-targeting agent that binds to the minor groove and crosslinks DNA, leading to DNA damage	Protease-cleavable linker	MedImmune LLC Gaithersburg, MD, U.S.
5	STI-6129 (anti-CD38 with Duostatin 5 site-specific C-LOCK conjugation)	IgG1	Duostatin 5.2 (Duo. 5.2) is a microtubule inhibitor derived from MMAF	Non-polyethylene glycol linker	Sorrento Therapeutics, Inc. San Diego, CA, U.S.
6	MT-0169 (anti-CD38 2nd generation engineered toxin body)	-	Genetically engineered de-immunized Shiga-like toxin-A-subunit (SLTA); results in ribosome inactivation	SLT-A genetically fused to antibody-like binding domains	Molecular Templates, Inc. Austin, TX, U.S.
7	Lorvotuzumab mertansine (IMGN901)	IgG1κ	Maytansine DM1, a microtubule inhibitor linked with an anti-CD56 antibody	Thiopentanoate linker (or reducible SPP (N-succinimidyl 4-(2-pyridyldithio)) linker)	ImmunoGen Waltham, MA, U.S.
8	STRO-001	IgG1	A p-azido-methyl-phenylalanine (pAMF)-containing anti-CD74 aglycosylated human IgG1; maytansinoid conjugated to two sites	A non-cleavable dibenzocyclooctyne linker	Sutro Biopharma, Inc. South San Francisco, CA, U.S.
9	ABBV-838 Azintuxizumab vedotin	IgG1κ	Monomethyl auristatin E (MMAE) conjugated to anti-CD2 subset 1 epitope (CS1) humanized recombinant IgG1κ antibody	Cleavable maleimidocaproyl-valyl-citrullinyl-p-minobenzyloxycarbonyl (mc-val-cit-PABC) linker	AbbVie North Chicago, IL, U.S.
10	Indatuximab ravtansine (nBT062)	IgG4	Maytansinoid drug DM4, a microtubule inhibitor, covalently conjugated to anti-CD138 chimerized IgG4 monoclonal antibody	Disulfide bond-based linker	Biotest Pharmaceuticals Corporation Boca Raton, FL, U.S.
11	DFRF4539A	IgG1	MMAE conjugated to anti-FcRH5 antibody	Cleavable linker	Genentech, Inc. South San Francisco, CA, U.S.

Abbreviations: BCMA, B-cell maturation antigen; IgG, Immunoglobulin G; DM1, mertansine; PBD, pyrrolobenzodiazepine; C-LOCK conjugation, method of linking antibody heavy and light chains following reduction in inter-chain disulfide bonds.

**Table 2:** Currently Active Clinical Trials of Antibody–Drug Conjugates in Multiple Myeloma.

Sl. No	ADC	Study Phase	Eligibility Criteria	Regimen Details	NCT Number
1	Anti-CD38 STI-6129.	Phase 1b/2a	Male/female, 18 years of age or older. Confirmed diagnosis of MM as defined by the IMWG. Relapsed/refractory myeloma.	Open-label, multicenter, dose-escalation study of STI-6129 intravenously (IV) administered once in a 4-week cycle. Seven dosing cohorts: 0.67 mg/kg, 0.88 mg/kg, 1.18 mg/kg, 1.56 mg/kg, 2.08 mg/kg, 2.77 mg/kg, 3.68 mg/kg.	NCT05308225
2	Anti-BCMA ADC (CC-99712).	Phase 1	Male/female, 18 years of age or older. Confirmed diagnosis of MM as defined by the IMWG. Relapsed/refractory myeloma.	Multicenter, open-label, dose finding study that includes: Experimental arm 1: CC-99712 monotherapy IV. Experimental arm 2: CC-99712 will be administered IV while BMS-986405 will be administered orally.	NCT04036461
3	Belantamab mafodotin (GSK2857916; BelaMaf) with lenalidomide + dexamethasone (Arm A), or with bortezomib + dexamethasone (Arm B) (DREAMM 6).	Phase 1/2	Male/female, 18 years of age or older. Confirmed diagnosis of myeloma. Relapsed/refractory myeloma.	Experimental arm A: BelaMaf + lenalidomide + dexamethasone. Experimental arm B: BelaMaf + bortezomib + dexamethasone.	NCT03544281
4	BelaMaf as monotherapy (Part 1) or as combination therapy (Part 2). Dose escalation will follow a 3 + 3 design.	Phase 1	Male/female, 20 years of age or older. Histologically/cytologically confirmed myeloma in a participant who: has undergone stem cell transplant or is transplant-ineligible; Part 1: has received at least 2 prior lines of anti-myeloma drugs containing at least 1 proteasome inhibitor and at least 1 immunomodulator; Part 2: has received at least 1 prior line of drugs; has demonstrated progression on, or within 60 days of the last therapy. Relapsed/refractory myeloma.	Open-label, dose-escalation study to investigate tolerability, safety, pharmacokinetics, immunogenicity and clinical activity of GSK2857916 in Japanese participants with relapsed/refractory MM. Experimental part 1: BelaMaf monotherapy administered IV. Experimental part 2, arm A: BelaMaf + bortezomib/ dexamethasone. Experimental part 2: arm B: BelaMaf + pomalidomide/ dexamethasone.	NCT03828292
5	Belantamab mafodotin.	Phase 2	Male/female, 18 years of age or older. Histologically/cytologically confirmed diagnosis of myeloma, and participant has undergone stem cell transplant or is transplant-ineligible and has failed at least 3 prior lines including an anti-CD38 antibody alone or in combination and is refractory to an immunomodulatory drug and to a proteasome inhibitor.	Open-label, randomized, two-arm study in participants with myeloma who had three or more prior lines of treatment, are refractory to a proteasome inhibitor and an immunomodulatory agent and have failed an anti-CD38 antibody (DREAMM 2). The study includes: Experimental: Frozen BelaMaf product at 2.5 mg/kg Experimental: Frozen BelaMaf product at 3.4 mg/kg. Experimental: Lyophilized BelaMaf product.	NCT03525678
6	Belantamab mafodotin. Study of carfilzomib, lenalidomide, dexamethasone and belantamab mafodotin in myeloma.	Phase 1/2	Male/female, greater than or equal to 18 years of age. Phase I: Relapsed or relapsed/refractory myeloma with 1–3 lines of prior therapy. Phase II: High-risk newly diagnosed symptomatic myeloma.	Phase I dose escalation and expansion study in RMM and RRMM followed by a single arm Phase II expansion in high risk, NDMM. Experimental Phase I: Carfilzomib, lenalidomide, dexamethasone, BelaMaf Experimental Phase II: Maximum tolerated dose from Phase I.	NCT04822337
7	Belantamab mafodotin as pre- and post-autologous stem cell transplant consolidation and maintenance.	Phase 2	Male/female, 18 years of age or older. Inclusion criteria—must have started therapy within 12 months of enrollment, received no more than two prior lines of induction therapy, with no prior progressive disease.	A Phase 2 study of BelaMaf in patients with myeloma prior to and following autologous stem cell transplantation (ASCT), in conjunction with lenalidomide maintenance. Experimental: BelaMaf at 2.5 mg/kg IV on day 42 relative to autologous stem cell infusion (day 0), on day +60, and every 90 days thereafter, for up to 2 years following ASCT.	NCT04680468
8	Belantamab mafodotin Platform study of belantamab as monotherapy and in combination with anti-cancer treatments in participants with relapsed/ refractory myeloma.	Phase 1/2	Male/female, greater than or equal to 18 years of age, histologically or cytologically confirmed diagnosis, having at least three prior lines of treatment including an immunomodulating agent, a proteasome inhibitor, and an anti-CD38 monoclonal antibody.	Phase I/II, randomized, open-label platform study of BelaMaf as monotherapy and with anti-cancer treatments in RRMM patients. The study includes: BelaMaf + GSK3174998 dose exploration BelaMaf + feladilimab dose exploration BelaMaf + nirogacestat dose exploration BelaMaf + dostarlimab dose exploration BelaMaf + isatuximab dose exploration BelaMaf + nirogacestat + lenalidomide + dexamethasone dose exploration BelaMaf + nirogacestat + pomalidomide + dexamethasone dose exploration Active comparator: BelaMaf monotherapy cohort expansion followed by Sub-study 1–7 cohort expansion.	NCT04126200
9	Belantamab mafodotin in myeloma participants with normal and varying degrees of impaired renal function (DREAMM12).	Phase 1	Male/female participants must be 18 years of age or older. (19 or older in the Republic of Korea). Must also have histologically or cytologically confirmed diagnosis, undergone autologous stem cell transplant (SCT) or is considered transplant-ineligible; has failed at least two prior lines of treatment, including an immunomodulatory drug and a proteasome inhibitor. In Republic of Korea, participants should also have relapsed or refractory disease after treatment with an anti-CD38 antibody.	DREAMM12 study includes: Experimental part 1: Participants with normal/mild impaired renal function (Normal: individual glomerular filtration rate [iGFR]: >=90 milliliter per minute; Mild impairment: iGFR: 60–89 mL/min); severe renal impairment (iGFR: 15–29 mL/min). BelaMaf 2.5 mg/kg IV Q3W on Day 1 of every 21-day cycle until progression, death, unacceptable toxicity, withdrawal of consent, or end of study. Experimental part 2: Participants with ESRD (iGFR: <15 mL/min), not on dialysis and on hemodialysis. BelaMaf either 2.5 mg/kg or 1.9 mg/kg (or other adjusted dose) IV Q3W on Day 1 of every 21-day cycle until progression, death, unacceptable toxicity, withdrawal of consent, or end of study. In Part 2, the dose will be decided after evaluation of pharmacokinetic and safety data of Part 1.	NCT04398745
10	Anti-CD74 ADC (STRO-001) Study of STRO-001, an anti-CD74 ADC, in patients with advanced B-cell malignancies.	Phase 1	Male/female, 18 years or older Confirmation of diagnosis Relapsed or relapsed/refractory disease.	The study includes IV infusion of STRO-001 on Day 1 of a 21-day cycle, until disease progression. Part 1: Dose-escalation study using an accelerated dose titration design. Part 2: Dose expansion study when Part 1 is completed. Enrollment will include separate tumor cohorts of myeloma and non-Hodgkin lymphoma.	NCT03424603
11	Belantamab mafodotin Study to investigate alternative dosing regimens of belantamab in relapsed or refractory myeloma (DREAMM 14).	Phase 2	18 years of age or older. Histologically or cytologically confirmed diagnosis and has undergone stem cell transplant or is transplant-ineligible and has failed at least three prior lines of therapy, including anti CD38 alone or in combination and is refractory to an immunomodulatory agent and a proteasome inhibitor.	The study includes: Cohort 1: Participants receiving BelaMaf at dose level (DL) 1 Cohort 2: Participants receiving BelaMaf at DL 2 Cohort 3: Participants receiving BelaMaf at DL 3 Cohort 4: Participants receiving BelaMaf at DL 4 Cohort 5: Participants receiving BelaMaf at DL4 with alternative dose modification.	NCT05064358

## Data Availability

Not applicable.

## Abbreviations

ADCC, antibody-dependent cellular cytotoxicity; ADCs, antibody–drug conjugates; ADCP, antibody-dependent cellular phagocytosis; AEs, adverse events; AL, light chain amyloidosis; BCMA, B-cell maturation antigen; BelaMaf, Belantamab mafodotin; CARs, chimeric antigen receptors; CDC, complement-dependent cytotoxicity; CR, complete response; DAR, drug:antibody ratio; DDR, DNA damage response; DM1, mertansine; DOR, duration of response; FCRL5, Fc-receptor-like 5; FcγR, Fcgamma receptor; FGFR3, Fibroblast growth factor receptor 3; FIH, first-in-human; ICAM, Intracellular adhesion molecule; ICD, immunogenic cell death; Igs, immunoglobulins; IMiD, immunomodulatory drug; IrRd, indatuximab ravtansine with lenalidomide and dexamethasone; IrPd, indatuximab ravtansine with pomalidomide and dexamethasone; LOT, lines of therapy; MHC, major histocompatibility complex; MMAE, monomethyl auristatin E; MMAF, monomethyl auristatin F; MRD, minimal residual disease; MTD, maximum tolerated dose; ORR, overall response rate; OS, overall survival; Pd, pomalidomide and dexamethasone; PFS, progression-free survival; PIs, proteasome inhibitors; RP2D, recommended Phase II dose; sBCMA, soluble BCMA; sCR, stringent complete response; SLAMF, Signaling lymphocyte activation molecule family member; TCR, triple-class refractory; TEAEs, treatment-emergent adverse events; TME, tumor microenvironment; VGPR, very good partial remission.
